# Synergistic Antibacterial Effect of Phage pB3074 in Combination with Antibiotics Targeting Cell Wall against Multidrug-Resistant Acinetobacter baumannii *In Vitro* and *Ex Vivo*

**DOI:** 10.1128/spectrum.00341-23

**Published:** 2023-06-01

**Authors:** Jun Luo, Libo Xie, Min Yang, Min Liu, Qianyuan Li, Peng Wang, Jinhong Fan, Jing Jin, Chunhua Luo

**Affiliations:** a The First College of Clinical Medical Science, China Three Gorges University, Yichang, China; b Yichang Central People's Hospital, China; C Yunnan Center for Disease Control and Prevention, Yunnan, China; University of Exeter

**Keywords:** phage-antibiotic synergy, multidrug-resistant *A. baumannii*, *in vitro*, *ex vivo*, mechanism

## Abstract

Synergistic effects of phages in combination with antibiotics have received increasing attention. In this present study, we isolated a new phage pB3074 against clinically isolated multidrug-resistant Acinetobacter baumannii. Phage pB3074 combined with cell wall-targeting antibiotics could produce synergistic antibacterial effect *in vitro* bactericidal activities. Further research indicates that the bacteriophage dose is critical to synergistic antimicrobial effect of phage and antibiotic combination. Cefotaxime and meropenem were selected as the representative cell wall-targeting antibiotics for further synergistic antibacterial study. Results illustrated that phage pB3074 and cefotaxime or meropenem combination was very effective for the removal of mature biofilm and inhibition of biofilm formation. In a pig skin explant model, results also showed that phage pB3074 and cefotaxime or meropenem combination was very effective for the treatment of wound infection *ex vivo*. Subsequent studies showed that some extent recovery of drug sensitivity to cell wall-targeting antibiotics might be vital mechanism of synergistic antibacterial effect between bacteriophage pB3074 and these antibiotics. The existence of antibiotics could promote phage adsorption and proliferation, which might also be potential mechanism for synergistic antibacterial activities and have been observed in cefotaxime and meropenem application. In summary, results in the current study demonstrated that phage pB3074 has the potential to be developed as an antibacterial agent and combined application of phages and antibiotics might be a new choice for the treatment of current multidrug-resistant bacterial infections.

**IMPORTANCE** Combined application of phages and antibiotics cannot only effectively inhibit the appearance of phage-resistant bacteria, but also reduce the effective use concentration of antibiotics, and even make some bacteria regain sensitivity to some resistant antibiotics. Therefore, phage-antibiotic combination (PAC) could improve the antibacterial activity of individual drug, providing a new choice for clinical treatment of multidrug-resistant bacterial infections.

## INTRODUCTION

Acinetobacter baumannii is global priority pathogen recommended by the World Health Organization (WHO) (1). The rise and prevalence of antimicrobial resistance in A. baumannii causes increasing difficulty to treat infections caused by them. Traditional antibiotic treatment is hard to meet the current treatment of drug-resistant bacteria infection, and new treatments for drug-resistant bacterial infections are need urgently. Phage-antibiotic combination treatments might be one promising approach (2).

Bacteriophage (phage) is a virus that could infect specific living host bacteria. Phage is an important agent for combating pathogenic bacteria in clinical treatments when it was initially discovered (3). The discovery of antibiotics made bacteriophage therapy gradually withdraw from the stage. In light of the increasing emergence of antibiotic-resistant bacteria, phage therapy has been recognized as a potential alternative or addition to antibiotics (4, 5). At present, reports on bacteriophage therapy in laboratory and clinical research are increasing, such as bacteriophage therapy for bone and joint infections, and bacteriophage therapy as a treatment option for transplant infections (6, 7).

Regrettably, bacteria also produce resistance to phages during phage therapy. The synergistic antibacterial effect produced by the combination of bacteriophages and antibiotics cannot only reduce the use concentration of antibiotics, but also inhibit the appearance of phage-resistant bacteria, which might be a good solution to the current bacteriophage therapy dilemma (8, 9). The antibacterial effect and mechanism of bacteriophage and antibiotic combinations have been more and more studied (10, 11).

The purpose of this study is to develop an effective biocontrol strategy based on bacteriophage and bacterial-resistant antibiotics combination against multidrug-resistant A. baumannii infection. In this study, we isolated and characterized a virulent phage pB3074 against a highly resistant isolate of A. baumannii 3074 (Bm3074). Further, the activity of bacteriophage pB3074 in combination with different antibiotics was investigated based on the antibacterial activity *in vitro*. The efficacy of bacteriophage pB3074 in combination with cefotaxime or meropenem in biofilm clearance and *ex vivo* skin explant wound infection model was evaluated. We also conducted a preliminary study on the synergistic mechanism between bacteriophage pB3074 and antibiotics.

## RESULTS

### Characterization and genome analysis of pB3074.

**FIG 1:**
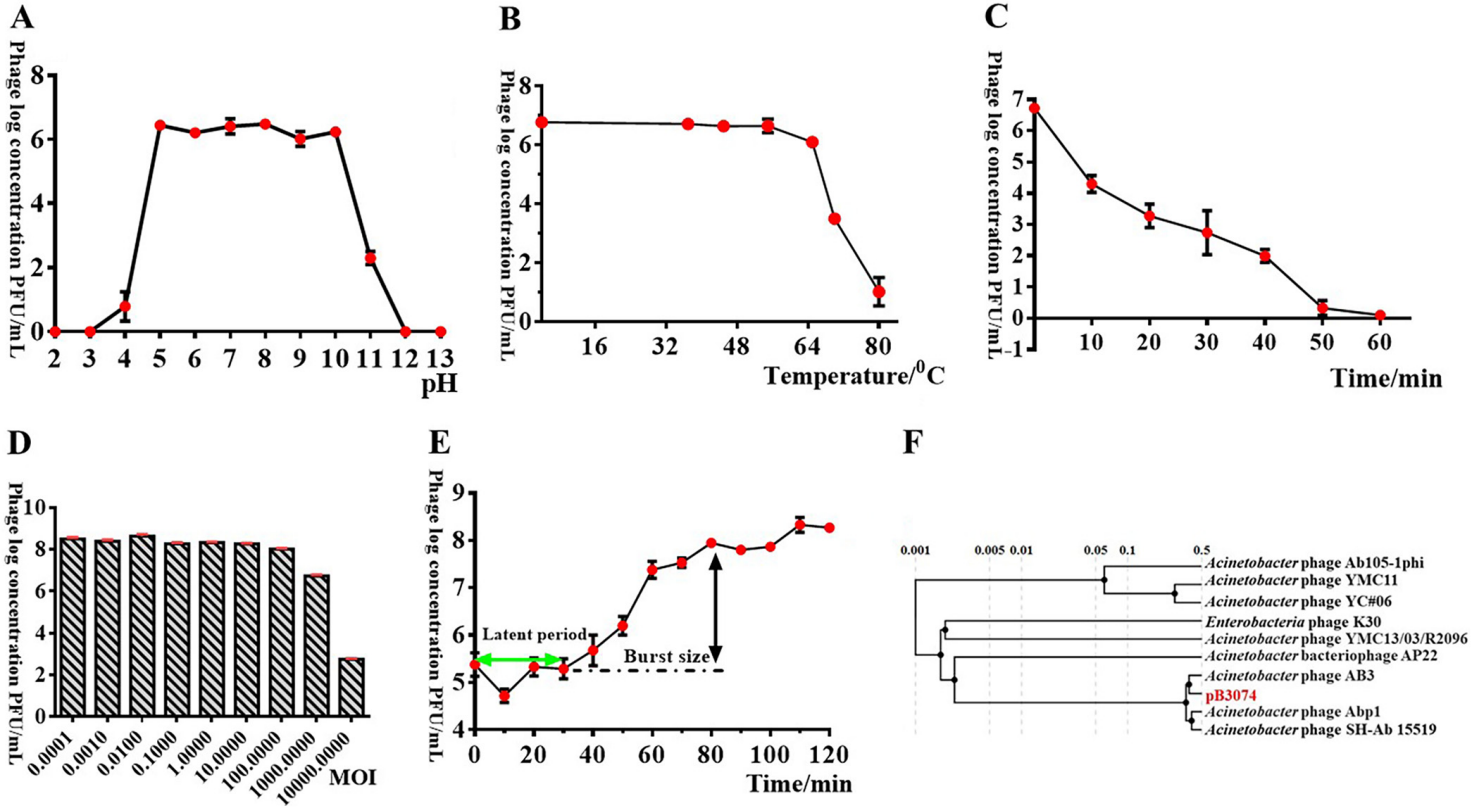
(A to E) Biological properties of phage pB3074. (A) pH sensitivity; (B) temperature tolerance; (C) UV tolerance; (D) determination of the optimal MOI; and (E) one-step growth curve. (F) Phylogenetic tree. Phage log concentration are calculated based on the double-layer agar method. Data are presented as the mean plus standard deviation (SD).

A total of 34 clinical A. baumannii strains and Escherichia coli, Pseudomonas aeruginosa, and Streptococcus pneumoniae were used followed by “plaque assay” to find out the host range of phagepB3074. pB3074 showed activity against 14 A. baumannii isolates, whereas no activity was observed against other strains used in this study (Table S10). Phage plaque diameter of pB3074 was measured as ~1 mm and has clear centers. Phage pB3074 maintained a good activity at pH values ranging from 5.0 to10, but its titers dramatically decreased about 4 log units at pH 4.0 and 11.0, respectively. When incubated at pH 3.0 and 12.0, the phage was completely inactive. Thus, the optimum pH range for phage pB3074 was found to be 5.0 to 10.0 (Fig. 1A). Phage pB3074 was survived steadily over a temperature range of 4°C to 65°C. When the phage was incubated at 70°C for 60 min, its log concentration of titer rapidly decreased to 3, and upon incubation at 80°C for 60 min, its log concentration of titer rapidly decreased to 1. These results indicated that phage pB3074 could tolerate a broader range of temperature environment (Fig. 1B). The titer of pB3074 decreased with the prolongation of UV irradiation. After 60 min of UV irradiation, all bacteriophages were inactivated, indicating that bacteriophages were sensitive to UV (Fig. 1C). There was no significant difference in ultimate yield of phage among various MOI ratios ranging from MOI = 0.0001 to MOI = 100. However, the highest bacteriophage titer had always been observed at MOI = 0.01. Based on the maximum phage titer obtained upon infection of B.m3074, the optimal MOI of phage pB3074 was 0.01 (Fig. 1D). The one-step growth curve of phage pB3074 showed a latent period of 30 min and a burst period of 50 min (Fig. 1E), followed by a plateau period. The size of the burst, calculated as the ratio of the average number of phages released per infected host cell before (2 × 10^5^ PFU/mL) and after the burst (1.90 × 10^7^ PFU/mL) was approximately 85 PFU/cell after 80 min (Fig. 1E).

The entire pB3074 genome was sequenced. After quality control, 50,851,676 reads were obtained. The final genome assembly yielded a 41,894 bp circular, dsDNA molecule with 39.38% G+C content, with no tRNAs identified. The total of protein coding genes was 51 (Table S11). The phylogenetic tree was constructed based on the whole-genome sequences of phage pB3074 and other phages (sequence accession number provided in Table S12). The results of the phylogenetic analysis showed that phage pB3074 was the most closely related to phage AB3, belonging to a member of a phiKMV-like virus in the T7 phage family (12) (Fig. 1F). We performed drug resistance gene analyses, and no drug resistance genes were found, suggesting that pB3074 might be potential for clinical therapeutic applications.

### Antibacterial activity and time-kill kinetics of the phage pB3074 and antibiotic combination under different doses of bacteriophage *in vitro*.

**FIG 2:**
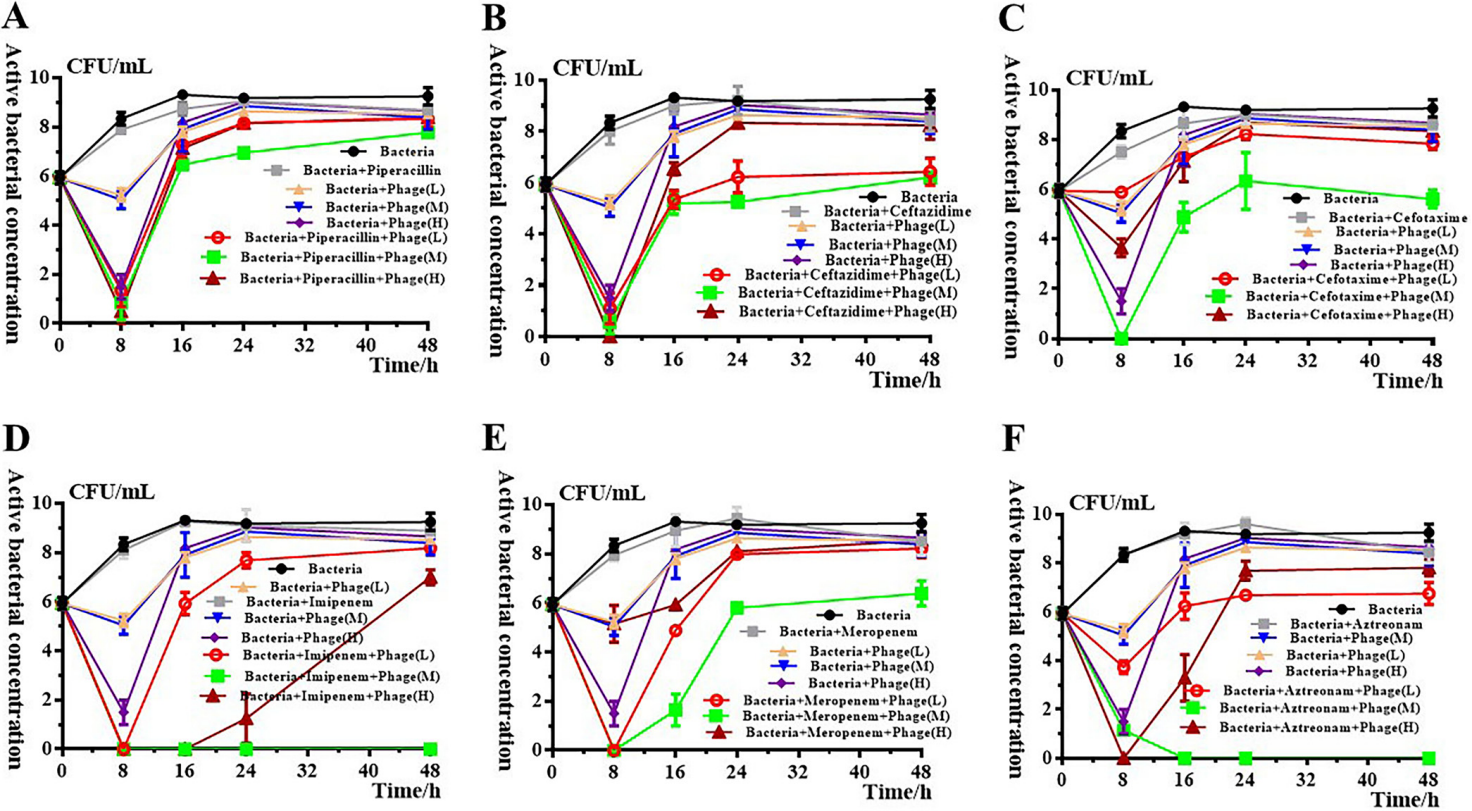
The effect of phage dose on the combination of phage pB3074 and cell wall-targeting antibiotics, including (A) piperacillin; (B) ceftazidime; (C) cefotaxime; (D) imipenem; (E) meropenem; and (F) aztreonam. The *y* axis represents the active bacteria amount, which is calculated by log (CFU/mL). The *x* axis represents culture time. Piperacillin, 128 μg/mL; ceftazidime, 16 μg/mL; cefotaxime,16 μg/mL; imipenem, 8 μg/mL; meropenem, 2 μg/mL and aztreonam, 8 μg/mL. L, low dose (MOI = 0.01); M, middle dose (MOI = 1); H, high dose (MOI = 100).

To confirm the possible synergistic activities of pB3074 with antibiotics, the antibiotics included were chloramphenicol, minocycline, piperacillin, ceftazidime, cefotaxime, ciprofloxacin, imipenem, gentamicin, meropenem, tetracycline, and aztreonam. Time-killing assays against Bm3074 illustrated that pB3074 combined with cell wall-targeting antibiotics, including piperacillin, ceftazidime, cefotaxime, imipenem, meropenem, and aztreonam, could produce synergistic antibacterial effect (Fig. 2). Furthermore, the synergistic antibacterial effect of piperacillin, ceftazidime, cefotaxime, imipenem, meropenem, and aztreonam, combined with different doses of bacteriophage were explored *in vitro*. As observed in the Fig. 2, synergistic antibacterial activity of antibiotic combined with phage at medium-dose (MOI = 1) and high-dose (MOI = 100) appear more obvious within initial 8 h. The synergistic antibacterial effect of antibiotic combined with phage at low-dose (MOI = 0.01) and medium-dos (MOI = 1) could be observed, even after 48 h of coculture, of which synergistic antibacterial effect at medium-does reveal stronger. The synergistic antibacterial effect of high-dose (MOI = 100) and antibiotic combination is further weakened and disappeared with the increasing incubation time. Based on these results, the phage dose, ranging from low-dose (MOI = 0.01) to medium-dose (MOI = 1) combined with antibiotic could have better synergistic antibacterial effect.

### Effect of the combination of pB3074 and antibiotic (cefotaxime or meropenem) *in vitro* activity against both biofilm formation and mature biofilm of Bm3074.

**FIG 3:**
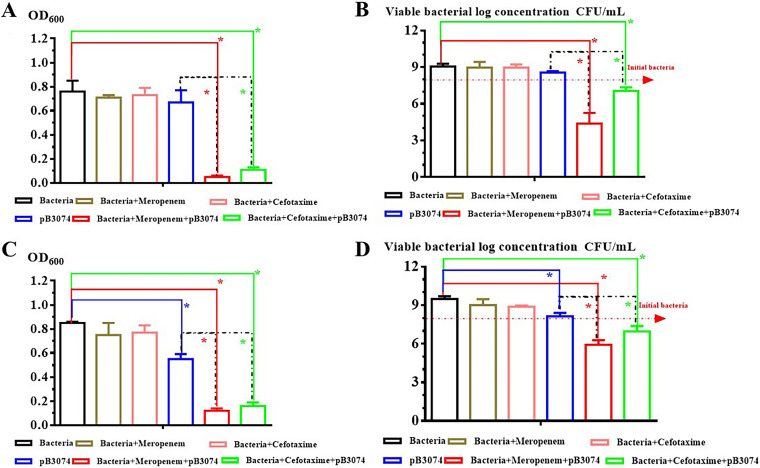
Evaluation of the combination of pB3074 and antibiotic *in vitro* activity against both (A, B) forming; and (C, D) mature biofilms of Bm3074. Antibiotics include cefotaxime (2×MIC) or meropenem (0.5×MIC). (A and C) Optical density at 600 nm (OD_600_) was measured using the microplate reader. (B and D) Viable bacteria counting was detected using dilution plate counting method. For the biofilm formation inhibition experiment in panels A and B, the phage and B.m3074, antibiotic and B.m3074, antibiotic, phage and B.m3074 were cocultured. For the mature biofilm-reducing experiment in panels C and D, phage, antibiotic, and antibiotic plus phage were added when mature biofilm was observed. Error bars represent SD of three independent experiments (*, *P* value < 0.05).

Effects of phage pB3074 and antibiotic (cefotaxime 2×MIC or meropenem 0.5×MIC) combination *in vitro* activity against both mature and forming biofilms of Bm3074 were evaluated by crystal violet assay and the viable bacterial count method. As shown in Fig. 3A and C, results crystal violet assay measured by a TECAN sunrise microplate reader illustrate that phage pB3074 alone could partly effective at eradicating mature biofilm but failed to prevent biofilm formation. Phage pB3074 and antibiotic (cefotaxime 2×MIC or meropenem 0.5×MIC) combination could effectively prevent biofilm formation and eradicate established biofilms. Identical results could be obtained from the dilution plate counting method (shown in Fig. 3B and D).

### The combination of phage and antibiotic limits bacterial regrowth in an *ex vivo* model of wound infection.

**FIG 4:**
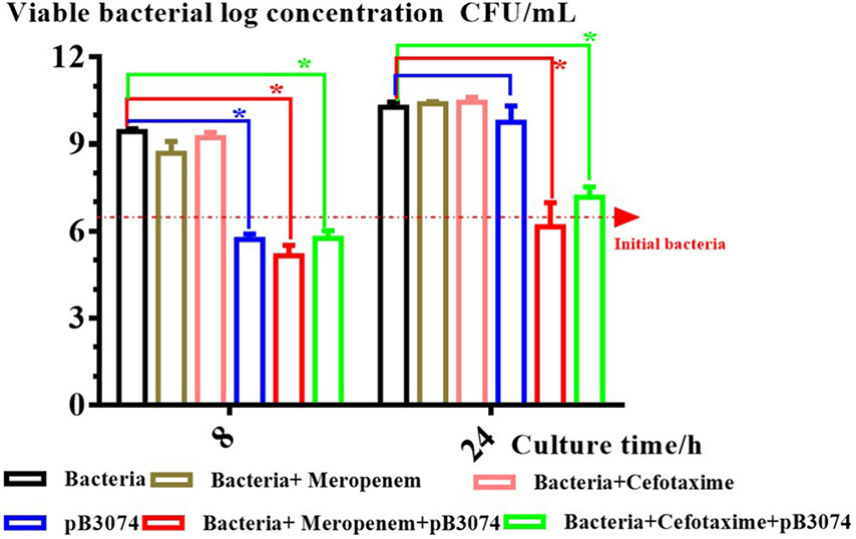
Antibacterial effect of pB3074 alone or in combination with antibiotic (cefotaxime [2×MIC] or meropenem [0.5×MIC]) in an *ex vivo* pig skin model of wound infection and treatment.

The antibacterial effect of phage and antibiotic alone or in combination against wound infections was investigated in an *ex vivo* pig skin model. Results (Fig. 4) show that, bacterial number in antibiotic (cefotaxime [2×MIC] or meropenem [0.5×MIC]) treatment group always almost identical to bacteria group throughout the whole treatment period. Compared with control group (bacteria group), cefotaxime (2×MIC) treatment group and meropenem (0.5×MIC) treatment group, bacterial number in treatment group, including phage alone, phage combined with antibiotic (cefotaxime [2×MIC] or meropenem [0.5×MIC]) have a significant reduction after 8-h posttreatment (*P* ≤ 0.05). Bacterial number in phage combined antibiotic (cefotaxime [2×MIC] or meropenem [0.5×MIC]) therapy still have a significant reduction after 24-h posttreatment (*P* ≤ 0.05). In contrast, bacterial number in bacteriophage-therapy (phage) alone have no significant difference with bacteria group after 24-h posttreatment.

### Susceptibility results of phage-resistant isolates.

According to the CLSI guideline (13), the antibiotic profiling of phage-resistant isolates were carried out by the standard broth microdilution method. These results show that phage-resistant isolates restore different levels of sensitivity to multiple antibiotics (Table 1; Fig. S2). In total, 27.27% (3/11) of phage-resistant isolates decreased its resistance to piperacillin by 4-fold (512 μg/mL to 128 μg/mL) and 36.36% (4/11) of phage-resistant isolates decreased its resistance to piperacillin by 8-fold (512 μg/mL to 64 μg/mL). Also, 36.36% (4/11) of phage-resistant isolates decreased its resistance to ceftazidime by 2-fold (64 μg/mL to 32 μg/mL) and 27.27% (3/11) of phage-resistant isolates decreased its resistance to ceftazidime by 4-fold (64 μg/mL to 16 μg/mL). In addition, 36.36% (4/11) of phage-resistant isolates decreased its resistance to cefotaxime by 16-fold (512 μg/mL to 32 μg/mL) and 32.36% (4/11) of phage-resistant isolates decreased its resistance to cefotaxime by 32-fold (512 μg/mL to 16 μg/mL). And, 32.36% (4/11) of phage-resistant isolates decreased its resistance to imipenem by 2-fold (64 μg/mL to 32 μg/mL), 9.09% (1/11) of phage-resistant isolates decreased its resistance to imipenem by 4-fold (64 μg/mL to 16 μg/mL), 45.45% (5/11) of phage-resistant isolates decreased its resistance to imipenem by 8-fold (64 μg/mL to 8 μg/mL), and 9.09% (1/11) of phage-resistant isolates decreased its resistance to imipenem by 16-fold (64 μg/mL to 4 μg/mL). Furthermore, 9.09% (1/11) of phage-resistant isolates decreased its resistance to meropenem by 2-fold (64 μg/mL to 32 μg/mL), 27.27% (3/11) of phage-resistant isolates decreased its resistance to meropenem by 8-fold (64 μg/mL to 8 μg/mL), 36.36% (4/11) of phage-resistant isolates decreased its resistance to meropenem by 16-fold (64 μg/mL to 4 μg/mL), 27.27% (3/11) of phage-resistant isolates decreased its resistance to meropenem by 32-fold (64 μg/mL to 2 μg/mL). Lastly, 27.27% (3/11) of phage-resistant isolates decreased its resistance to aztreonam by 2-fold (64 μg/mL to 32 μg/mL), 54.54% (6/11) of phage-resistant isolates decreased its resistance to aztreonam by 4-fold (64 μg/mL to 16 μg/mL), and 18.18% (2/11) of phage-resistant isolates decreased its resistance to aztreonam by 8-fold (64 μg/mL to 8 μg/mL).

**TABLE 1:** Drug sensitivity results of Bm3074 and phage-resistant isolates

Strain no.	Type^*a*^	Pip^*b*^	Cef	Cefo	Imi	Mer	Aztr
pB3074	Wild	512/R	64/R	512/R	64/R	64/R	64/R
pB3074_tD1	Resistant	512/R	32/R	16/I	4/S	2/S	16/I
pB3074_tD2	Resistant	128/R	32/R	512/R	8/I	8/I	16/I
pB3074_tD3	Resistant	512/R	64/R	512/R	8/I	32/R	8/S
pB3074_tD4	Resistant	512/R	64/R	32/I	8/I	4/S	16/I
pB3074_tD5	Resistant	128/R	64/R	32/I	32/R	2/S	32/R
pB3074_tL3-1	Resistant	64/I	32/R	16/I	8/I	4/S	8/S
pB3074_tL3-2	Resistant	64/I	16/I	32/I	8/I	4/S	16/I
pB3074_tL5-1	Resistant	128/R	64/R	16/I	32/R	8/I	32/R
pB3074_tL5-2	Resistant	64/I	16/I	16/I	32/R	8/I	32/R
pB3074_tL1	Resistant	64/I	32/R	512/R	16/R	2/S	16/I
pB3074_tL4	Resistant	512/R	16/I	32/I	32/R	4/S	16/I

a Resistant (Type) indicates that isolate is resistant to phage pB3074. MIC is chosen according to the Clinical and Laboratory Standards Institute guidelines. Susceptible (S) indicates that bacteria are sensitive to conventional doses of drugs. Intermediate (I) indicates that bacteria might be sensitive to antibiotics that require high doses or antibiotics may be effective for infections in drug concentrated areas of the body. Resistant (R) indicates that bacteria are resistant to conventional doses of drugs.

b Antibiotic concentration (μg/mL). Piperacillin (Pip) MIC,16 μg/mL; Cefotaxime (Cef) MIC, 8 μg/mL; Cefoxime (Cefo) MIC, 8 μg/mL; Imipenem (Imi) MIC, 4 μg/mL; Meropenem (Mer) MIC, 4 μg/mL; Amtreonam (Azt) MIC, 8 μg/mL.

### Effect of antibiotics on bacteriophage proliferation.

**FIG 5:**
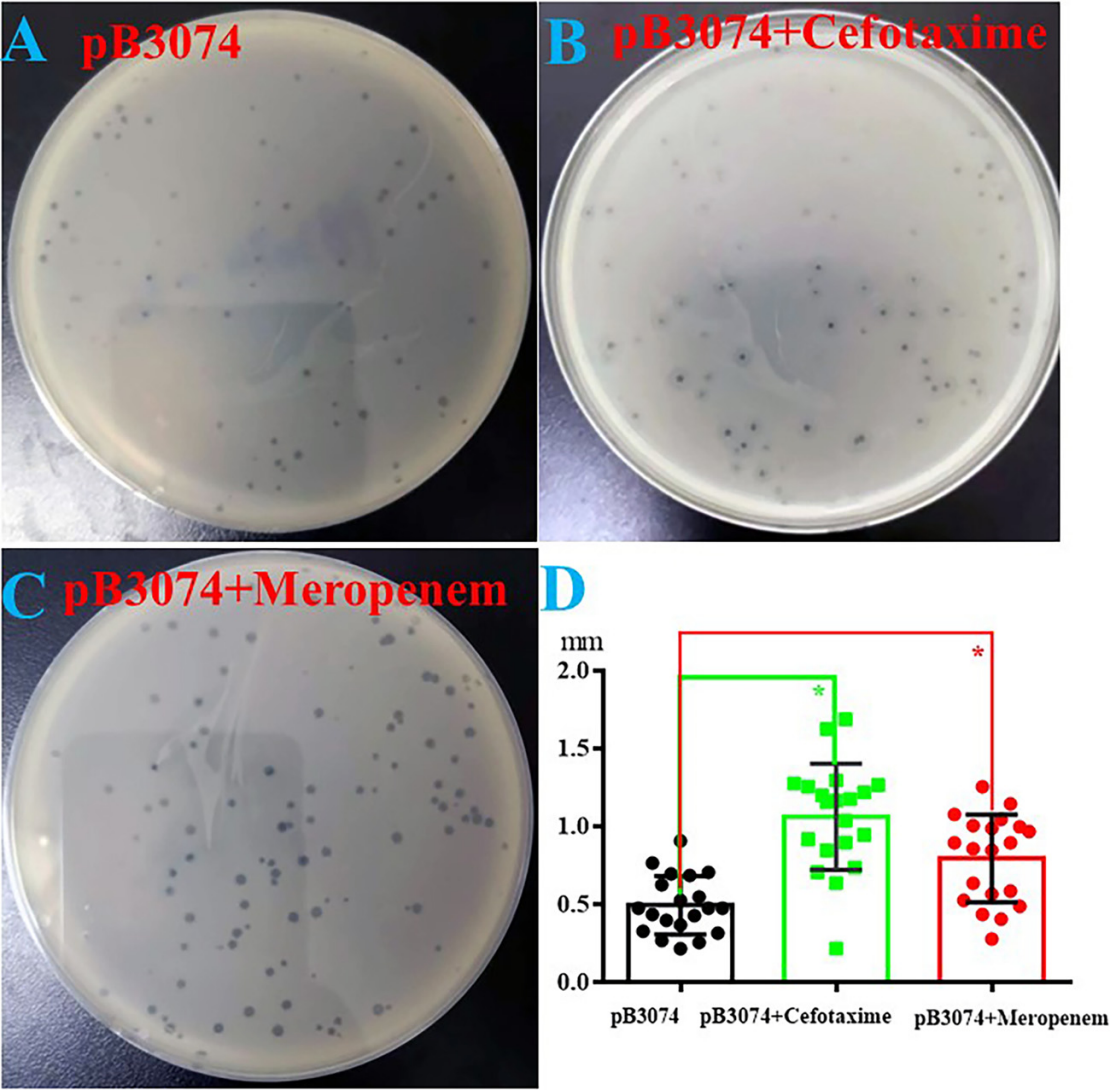
Bacteriophage proliferation with and without antibiotic (A to C) and diameter of bacteriophage plaque measured and statistically compared (D). Cefotaxime (2×MIC) or meropenem (0.5×MIC) used in this study.

Results (Fig. 5) showed that the size of halo, in the presence of cefotaxime (2×MIC) or meropenem (0.5×MIC), is 1.07 ± 0.26 mm or 0.80 ± 0.24 mm, respectively. The size of halo, in the absence of antibiotic, is 0.50 ± 0.15 mm. Compared with the phage cocultured with the host bacteria alone, presence of cefotaxime (2×MIC) or meropenem (0.5×MIC) made the overall phage plaque significantly larger.

## DISCUSSION

In this study, we isolated bacteriophage pB3074 against multidrug-resistant A. baumannii with strong temperature tolerance and wide pH adaptation range. After the whole-genome sequencing analysis, we performed drug resistance gene analyses, and no drug resistance genes were found, suggesting that pB3074 might be potential for clinical therapeutic applications. Phylogenetic tree (Fig. 1F) shows that phage pB3074 is the closest to A. baumannii phage AB3 but there are still great differences (Fig. 1F; Fig. S3) based on sequence homology analysis results generated with software MAUVE. The homology search between phage pB3074 and phage AB3 revealed a percentage cover of 49.1% and a percentage identity of 93.5%, which indicates that pB3074 is a new phage. We speculate that phage pB3074 genome is circular based on the consistency of the first and last frames of the genome sequence. However, the genome structure (linear or circularly permuted) should be confirmed in further experiments.

It is interesting that bacteriophage pB3074 and a series of cell wall-targeting antibiotics could produce different levels of synergistic effects *in vitro*. At present, there have been many reports on the synergistic effects of bacteriophages and some antibiotic *in vitro*, but the synergistic effects of such bacteriophage and a series of cell wall-targeting antibiotics are rarely discussed (14, 15).

The dosage of phage is important for synergistic antibacterial activity between phages and antibiotics *in vitro*. As shown in the results (Fig. 2), high-dose bacteriophages exhibited superior synergistic antibacterial activity during the initial 8-h culture period. However, with continuous coculture, bacteria began to develop resistance to phage under high doses of phages. The impact of phage dose on bacteriophage-antibiotic combination therapy have been noted, and development of bacterial resistance to phages under the high titer bacteriophage might have originated from phages prey pressure (8, 10). The underlying mechanism still is unclear and remains to be solved.

For biofilm-forming pathogenic bacteria, biofilm formation adds difficulty to treat clinical infection. A. baumannii is easy to form biofilms in many colonization places (16). As shown in Fig. 3, bacteriophage pB3074 and ceftazidime or meropenem combination could be effective for cleaning mature biofilm and preventing biofilm formation. Studies have shown that combined antibiotic phage treatment could be effective for prevention and control of biofilms (17, 18). Thus, the combination of bacteriophages and antibiotics might be a new and developable strategy to combat infections from biofilm-forming bacteria in clinical.

Experiments on tissue explants could illustrate possible clinical therapeutic applications of antibiotic and phage combination. In this study, we used pig skin as the explant to carry out the experiment, and results (Fig. 4) indicated that the combination of bacteriophage and antibiotic had a good therapeutic effect on the wound infection. Other experiments based an *ex vivo* pig skin model of wound infection also have illustrated that bacteriophage and antibiotic combination could be effective for treatment of skin wound infections (19, 20). Therefore, bacteriophage and antibiotic combination have potential clinical application in *ex vivo* tissue-based infection.

The antibiotic category in clinical applications of bacteriophage and antibiotic combination are initially adopting bacterial sensitive antibiotic and the combination of bacteriophages and bacterial-resistant antibiotics are relatively few. In this study, we showed that bacteriophage pB3074 and a series of cell wall-targeting antibiotics combination could produce good synergistic antibacterial effect. We preliminarily explored the synergistic bactericidal mechanism and results show that bacteriophage-resistant bacteria have recovered different degrees of drug sensitivity to a series of cell wall-targeting antibiotics (shown in Table 1; Table S1), which have been illustrated in other papers (11, 21). Based on our ongoing research, we speculate that sites implicated in phage resistance are associated with bacterial antibiotic resistance and the impact of sites on antibiotic resistance are different, which might lead to differences in the recovery of sensitivity of phage-resistant bacteria to various cell wall-targeting antibiotics. Based on double-halo effect illustrated in Fig. 5B, pB3074 endolysin that belongs to glycoside hydrolase family based on the speculation from phage genome sequence, as reported in the current literature (22), might increase cell wall permeability by cleaving a sufficient number of peptidoglycan bonds, which further enhances antibiotic penetration. However, definite mechanism of synergism is still unclear. We observed the phage plaque formation in coculture of phage with cefotaxime or meropenem, and found that the presence of cefotaxime or meropenem could promote phage proliferation. Some studies have illustrated that whether bacteria are resistance to a given antibiotic, antibiotics might affect bacterial morphology, thus promoting phage proliferation by enhancing phage adsorption (15, 23).

In conclusion, the main finding of the study indicates that phage–antibiotic combination could effectively improve the antibacterial activity of individual drug, providing a new choice for clinical treatment of multidrug-resistant bacterial infections.

## MATERIALS AND METHODS

### Bacterial strains and growth conditions.

Bm3074 as the host bacteria, isolated from bronchoalveolar lavage fluid in Yichang Central People’s Hospital (Hubei Province, China), was used in the study. Approximately 34 clinical A. baumannii isolates, P. aeruginosa, S. pneumoniae, and E. coli, isolated from the Yichang Central People’s Hospital (Hubei Province, China), were also used in the current study. These strains preserved in 25% glycerol at 80°C were routinely streaked onto Luria Bertani agar plates (LB; 10 g of NaCl, 5 g of yeast extract, 10 g of tryptone, and 15 g of agar per L, where yeast extract, tryptone, and agar were purchased from Oxoid Ltd., Basingstoke, Hampshire, England) and incubated overnight at 37°C. After picking a single colony on the plate into 5 mL fresh LB broth, the bacteria were grown about 8 to 12 h at 37°C and 200 rpm. The bacterial suspension was centrifuged at 6,000 rpm for 5 min. After discarding the supernatant, the bacterial pellet was resuspended in 1 mL fresh LB broth. The determination of the bacteria CFU was based on the plate dilution method and serial dilutions of the bacteria were prepared for the subsequent experiments. Antimicrobial profiling of Bm3074 and other isolates was performed by using the broth microdilution method (10).

### Phage isolation, propagation, and purification.

Bacteriophage pB3074 specific to Bm3074 was isolated directly from a domestic sewage sample collected in Yichang, Hubei Province, China. The phage was isolated using the phage enrichment technique as described in our previous study with slight modifications (10). Briefly, 1 mL of the log-phase host bacteria Bm3074, 75 mL of double-LB broth, and 75 mL of the filtered water samples were added to a 250-mL triangular flask and co-incubated at 37°C and 150 rpm for 24 h. After incubation, the mixture was centrifuged at 10,000 rpm for 10 min, and the supernatant was filtered through a 0.22-mm membrane filter (Millipore Sigma, Bedford, MA). The presence of phage was confirmed using a double-agar overlay method (24). Then, a single plaque was picked, resuspended in SM buffer (50 mM Tris-HCl, pH 7.5, 150 mM NaCl, 10 mM MgCl_2_, and 2 mM CaCl2), vortexed, and centrifuged. The supernatant was serially diluted and incubated with host bacteria. The phage was purified as described above. The process was repeated three times, ensuring that plaque morphology remained the same during the iterative process. The phage isolated against Bm3074 was designate pB3074. A high-titer phage lysate was prepared by overnight propagation in a log-phase bacterial culture with shaking (180 rpm) at 37°C. The phage lysate was subsequently centrifuged at 10,000 rpm for 10 min at 4°C, and the supernatant was filtered through a 0.22-mm membrane filter. The phage lysate was preserved at 4°C for further experiments. The phage titer was determined by means of a double-agar overlay assay as previously described (24).

### Bacteriophage characterization.

**(i) Host-range specificity tests.** A total of 34 clinical A. baumannii isolates, E. coli, P. aeruginosa, and S. pneumoniae, were used to assess the host range of the isolated bacteriophage. Host range analysis based on the standard spot test (10). Briefly, 100 μL of bacterial culture were inoculated into 5 mL of molten LB top agar and overlaid onto LB agar plates. Each overlay was allowed to solidify for 15 min. Five μL of the phage cultures of about 10^8^ PFU/mL were dropped onto the overlaid top agar. After 12 h of culturing at 37°C, the presence or absence of a lysis zone was observed. Spot tests were repeated in triplicate

**(ii) pH and thermal stability.** pH and thermal stability were analyzed based on our previous method (10). For pH stability test, 100 μL of phage solution (about 10^6^ PFU/mL) was mixed with 900 mL of physiological saline (pH adjusted with NaOH or HCl, ranging from 2 to 13). These mixtures were incubated at 37°C for 2 h, and the phage survival rate was determined using the double-layer agar method. For thermal stability tests, 2 mL of phage solution (about 10^6^ PFU/mL) was incubated at 4°C, 37°C, 45°C, 55°C, 65°C, 70°C, and 80°C for 60 min. The phage survival rate was determined using the double-layer agar method. All tests were performed in triplicate, and the error bar represents standard error of the mean.

**(iii) Phage UV survival assay.** Five mL of bacteriophage solution (3 × 10^6^ PFU/mL) is poured into the culture dish and the culture dish was then exposed to UV irradiation (254 nm). Two-hundred microliter samples were taken out at 10-min intervals during incubation and were immediately diluted to determine the phage titer by using the double-layer agar method. The experiment was repeated in triplicate, and the error bar represents standard error of the mean.

**(iv) Multiplicity of infection.** To determine the optimal multiplicity of infection (MOI), 100 μL of phage solution (4 × 10^5^ PFU/mL) and 100 μL of serially diluted pB3074 host culture (10^1^, 10^2^, 10^3^, 10^4^, 10^5^, 10^6^, 10^7^, 10^8^, and 10^9^ CFU/mL) were mixed with 5 mL of LB liquid medium and cocultured. After incubation for 3 h at 37°C, the cells were pelleted by centrifugation at 12,000 g for 10 min at 4°C, and the supernatant was filtered by a 0.22-mm membrane filter. The phage titers were measured by the double-layer agar method. The phage/bacterium ratio at which the highest phage titer could be obtained was considered the optimal MOI.

**(v) One-step growth curve.** A one-step growth curve of the phage was performed as previously described (10). Briefly, exponential-phase Bm3074 was centrifuged at 6000 × *g* for 20 min at 4°C. After discarding the supernatant, the pelleted cells were resuspended in LB media. The host bacteria were infected with pB3074 at MOI of 0.01 (phage/bacterium ratio, 10^5^ PFU/mL to 10^7^ CFU/mL) and were allowed to adsorb for 5 to 10 min at 37°C. For the removal of unabsorbed bacteriophages, the mixture was centrifuged at 12,000 g for 30 s. Then, the pellet was added to LB media (5 mL) and incubated at 37°C. Two-hundred-microliter samples were taken out at 10-min intervals during incubation and were immediately serially diluted to determine the phage titer by using the double-layer agar method. The experiment was repeated in triplicate, and the error bar represents standard error of the mean.

**(v) Phage DNA extraction and sequencing.** Phage DNA extraction and sequencing were based on our previous description (10). Fresh phage solution was centrifuged at 12,000 rpm for 10 min at 4°C, and the supernatant was filtered by a 0.22-mm membrane filter. DNase I and RNase A were added to the above-described filtrated phage solution in order to remove free nucleic acids and viral proteins. Then, phage DNA was extracted using the TIANamp virus DNA/RNA kit (DP315, Tiangen Biotech [Beijing] Co., Ltd.) based on the reagent instruction. Phage genome sequencing was performed by Sangon Biotech (Shanghai) Co., Ltd., China. Genomes were sequenced with Illumina HiSeq. In total, 50,851,676 reads were generated, and raw reads were trimmed to eliminate low-quality reads and adaptor sequences using Trimmomatic (version 0.36) (25). Clean reads were used for *de novo* assembly with SPAdes (version 3.5.0) (26). Genome annotation using Prokka (version 1.1.0) (27) was carried out by the sequencing service provider. Genes were identified by Prokka. Further gene annotation was conducted via NCBI BLAST+ (28) and aligned to several protein databases, including CDD (29) NR (http://ncbi.nlm.nih.gov/), Swiss-Prot (30), TrEMBL (30), GO (31) and KEGG (32). When the annotation results were inconsistent from different database, then aligned results of NR database were selected, followed by the Swiss-Prot, TrEMBL, CDD, GO, and KEGG databases (33).

In addition, phage genomic sequences were checked for genes coding antibiotic resistance at the website https://card.mcmaster.ca/. The phylogenetic tree was constructed from the multiple-genome sequence alignment using ViPTree: https://www.genome.jp/viptree.

### Antibacterial activity and time-kill kinetics of the phage pB3074 and antibiotic combination under different doses of bacteriophage *in vitro*.

The effect of phage dose on the combination of phage pB3074 and cell wall-targeting antibiotics, including piperacillin, ceftazidime, cefotaxime, imipenem, meropenem, and aztreonam, was assessed in 96-well plates based on the bacterial growth tracking. One-hundred microliter aliquots of dilute bacterial suspensions (2.5 × 10^5^ CFU/mL) were added to a 96-well plate. For control group, 100 μL of fresh LB medium were added to wells. For the experiment group, 100 μL of antibiotic (piperacillin, ceftazidime, cefotaxime, imipenem, meropenem, or aztreonam), 100 μL of phage solutions at three dose levels ranging from low dose (MOI = 0.01), middle dose (MOI = 1), and high dose (MOI = 100), 100 μL of phage solutions at above three dose levels and antibiotic (piperacillin, ceftazidime, cefotaxime, imipenem, meropenem, or aztreonam) in combination were added. The concentrations of the antibiotics used could be found in Table S1. These 96-well plates were incubated at 37°C for 24 h, and viable bacterial counts were determined after 0 h, 8 h, 16 h, 24 h, and 48 h of incubation using the agar dilution plate counting method (34). These plates were incubated at 37°C overnight and bacterial colonies were counted. The experiment was done in triplicate, and the error bar represents standard error of the mean. Synergy is defined as a 2-log_10_-CFU/mL kill compared to the most effective agent (or double-combination regimen) alone at 24 h. Bactericidal activity is defined as a 3-log_10_-CFU/mL reduction from baseline (35).

### Evaluation of the combination of pB3074 and antibiotic *in vitro* activity against both biofilm formation and mature biofilm of Bm3074.

Based on the effective antibiotic used in combination with specific bacteriophage (Table S1), we have selected meropenem and cefotaxime as representatives of cell wall-targeting antibiotics in subsequent study. Antibiofilm activity of phage pB3074 and antibiotic (cefotaxime or meropenem) combination was assessed as previously described (10), and antibiotic concentration chosen was also based on Table S1. Briefly, overnight cultures of Bm3074 were diluted 1:100 (vol/vol) in fresh TSB medium (Beijing Solar Bio Science & Technology Co., Ltd.). Then, 400 μL of bacterial suspension alone, 400 μL of bacterial suspension treated with antibiotic, including cefotaxime (2×MIC) or meropenem (0.5×MIC) alone, 400 μL of bacterial suspension cultured with phage (about 10^6^ PFU/mL) alone or in combination with cefotaxime (2×MIC) or meropenem (0.5×MIC), were inoculated into a 24-well polystyrene microtiter plate (BKMAM), and these plates were incubated for 24 h at 37°C. Total biomass was quantified by performing the crystal violet staining assay as described previously (19). Briefly, after washing the biofilm with PBS, 1 mL of 1% (wt/vol) crystal violet was added to each well. 5 min later, the excess of crystal violet was removed by washing twice with ultra-pure double distilled water. The remaining dye was then solubilized by adding glacial acetic acid (33%; Merck) and ethanol (2:8) and the absorbance at 595 nm (A595) was measured using a TECAN sunrise microplate reader.

Effect of phage pB3074 and antibiotic combination on mature biofilm reducing was also studied. Overnight cultures of Bm3074 were diluted 1:100 (vol/vol) in fresh TSB medium. Then, 400 μL of bacterial suspension was inoculated into a 24-well polystyrene microtiter plate (BKMAM), and the plates were incubated for 24 h at 37°C. Afterward, the planktonic phase was removed, and the biofilms were washed twice with phosphate-buffered saline (PBS; 137 mM NaCl, 2.7 mM KCl, 10 mM Na2HPO4, 2 mM KH2PO4 [pH 7.4]). The remaining adhered cells were then treated with 0.4 mL of TSB medium alone or using the same medium with cefotaxime (2×MIC) or meropenem (0.5×MIC) alone, phage (about 1.2 × 10^8^ PFU/mL) alone or in combination with cefotaxime (2×MIC) or meropenem (0.5×MIC) at 37°C. Treatment was allowed to act for 6 h. Then, the planktonic phase was removed and the adhered phase was washed twice with PBS. Total biomass was quantified by performing the crystal violet staining assay as described above.

To assess the efficacy of the different treatments, the number of viable attached cells were also quantified. The number of viable cells present in the biofilms were determined by using the spot test (10). Briefly, biofilms were scraped and resuspended in PBS. Afterward, 10 μL droplets from 10-fold serial dilutions of this cell suspension were spotted onto LB agar plates and allowed to dry. These plates were then incubated at 37°C for 24 h and counted.

### An *ex vivo* pig skin model of wound infection and treatment.

To assess the antibacterial effect of pB3074 and antibiotic combination on a biotic surface, the pig skin model was used based on previous study with minor modification (20). Pig skin used in the study was disinfected with 75% ethanol. Next, the skin was cut in 1.5 × 1.5 cm explants. To mimic a wound, a wound bed of about 50 mm diameter and 1 mm depth was made using a knife in specific explants. Next, all the explants were submerged in 75% ethanol for 1 h followed by 1 h of UV decontamination to ensure complete sterility. Overnight Bm3074 bacterial culture (6.50 × 10^8^ CFU/mL) was diluted 100 times with fresh LB medium and cultured to exponential growth phase. Fifty-microliter aliquots of bacteria culture was added to each wound area. All the explants were placed in 24-well plates containing 1 mL physiological saline agar (0.9% (wt/vol) NaCl, 0.5% (wt/vol) agar, pH 5.5) to mimic human skin conditions and incubated at 37°C for 2 h to allow the bacteria to establish an infection. Then, 50 μL of PBS, 50 μL of antibiotic (cefotaxime [2×MIC] or meropenem [0.5×MIC]), 50 μL of phage solution or 50 μL of phage solution plus antibiotic (cefotaxime [2×MIC] or meropenem [0.5×MIC]) were applied to the infected wounds. These plates were incubated for additional 24 h at 37°C. Then, the bacteria were harvested by adding 1,000 μL PBS to the wound area with gentle rubbing using a plastic loop. The treatment outcomes of different groups were evaluated by the viable bacterial count method. The experiment was performed in biological replicates (*n* = 3).

### Isolation and antimicrobial profiling of phage-resistant isolates.

These phage-resistant isolates were isolated as previously described (21). Exponential-phase clinically isolated Bm3074 was mixed with the phage supernatant at MOI =100. After incubation at 37°C for 24 h, the bacterial suspension was diluted in LB and then spread on LB plates followed by incubation at 37°C overnight. Single colonies were isolated and streaked on LB plates for purifying a single and pure colony. A spot test was used to confirm the phage-resistant isolates. Antimicrobial profiling of Bm3074 and other phage-resistant isolates were performed by the broth microdilution method.

### Effect of antibiotics on bacteriophage proliferation.

Effect of antibiotics on bacteriophage proliferation was studied by double-layer culture method. Phage pB3074 (about 10^6^ PFU/mL) is cocultured with the host bacteria Bm3074 (about 10^6^ CFU/mL) alone or mixed with antibiotic (cefotaxime [2×MIC] or meropenem [0.5×MIC]). These plates were placed at 37°C for 24 h. Phage plaque diameters were measured with Adobe Photoshop CS5. Petri dish was divided into four sections and five phages with different sizes in each section were selected for plaque diameter measurement.

### Statistical analysis.

Experimental data acquired in this study were analyzed based on GraphPad Prism software version 6.0 (GraphPad Software Inc., USA). Significance difference was analyzed by two-sample *t* test. Differences with *p* < 0.05 were considered significant at 95% confidence interval.

### Ethical approval.

The study was approved by the Ethics Committees of Yichang Central People’s Hospital (2019BA210).

### Data availability.

The data that support the findings of this study are found in the supplemental materials (Table S1 to Table S12; Fig. S1 to Fig. S3). The genomic database of pB3074 is available at the URL https://pan.baidu.com/s/1IxBkmjwXVxZonLL0Y5fDYw?pwd=zcbm (Code: zcbm).
