# Supplementary material for: Synergistic Antibacterial Effect of Phage pB3074 in Combination with Antibiotics Targeting Cell Wall against Multidrug-Resistant Acinetobacter baumannii In Vitro and Ex Vivo
Source: Microbiol Spectr. 2023 Jun 1;11(4):e00341-23. doi: 10.1128/spectrum.00341-23 (PMC10434185; doi:10.1128/spectrum.00341-23)
Supplement: Supplemental file 1 — Supplemental material. Download spectrum.00341-23-s0001.pdf, PDF file, 0.4 MB [file spectrum.00341-23-s0001.pdf]

Supplementary materials

Synergistic antibacterial effect of phage pB3074 in combination with antibiotics targeting cell wall against multi-drug resistant *Acinetobacter baumannii in vitro* and *ex vivo*

Running title

Phage pB3074 and antibiotic synergy *in vitro* / *ex vivo*

Jun Luo[a,b #+], Libo Xie [a,b+], Min Yang[c+], Min Liu[a, b], Qianyuan Li[a, b], Peng Wang[a, b], Jinhong Fan [a,b], Jing Jin[a,b#], Chunhua Luo[a,b#]

[a] The First College of Clinical Medical Science, China Three Gorges University, Yichang, China

[b] Yichang Central People's Hospital, China

[c]Yunnan Center for Disease Control and Prevention, Yunnan, China

[+] These authors contribute equally to this work

[#]To whom correspondence should be addressed.

Jun Luo Email: lj1988cby@126.com Tel/Fax: +86 0717-6483519

Jing Jin Email: 357601801@qq.com Tel/Fax: +86 0717-6483519

Chunhua Luo Email: lchlgj2004@aliyun.com Tel/Fax:+86 0717-6483519

Table S1 Effective antibiotic concentration used alone or in combination with bacteriophage against B.m#3074 in current study

Antibiotic Type	Pip	Cef	Cefo	Imi	Mer	Azt
B.m#3074+Antibiotic	512	64	**512**	64	**64**	64
pB3074+Antibiotic+B.m#3074	128	16	**16**	8	**2**	8
Antibiotic reduction folds	4	4	**32**	8	**32**	8

Note: Antibiotic concentration(μg/mL). MIC (Minimum inhibitor concentration, MIC) selection based on the American Association for Clinical and Laboratory Standards. Piperacillin (Pip) MIC: 16μg/mL, Cefotaxime (Cef) MIC: 8 μg/mL, Cefoxime (Cefo) MIC: 8 μg/mL, Imipenem (Imi) MIC: 4μg/mL, Meropenem (Mer) MIC: 4 μg/mL, Amtreonam (Azt) MIC: 8 μg/mL.

Table S2 Phage pB3074 pH sensitivity raw data

**pH**	**Triple Repeat Results**			**Average**	**Standard deviation**
2.00	0.00	0.00	0.00	0.00	0.00
3.00	0.00	0.00	0.00	0.00	0.00
4.00	0.60	1.48	0.30	0.79	0.46
5.00	6.28	6.65	6.40	6.44	0.06
6.00	6.16	6.48	6.00	6.21	0.08
7.00	6.48	6.74	6.00	6.41	0.24
8.00	6.51	6.62	6.30	6.48	0.10
9.00	5.81	6.00	6.27	6.02	0.23
10.00	6.09	6.40	6.23	6.24	0.07
11.00	2.30	2.70	1.90	2.30	0.20
12.00	0.00	0.00	0.00	0.00	0.00
13.00	0.00	0.00	0.00	0.00	0.00

Table S3 Phage pB3074 Temperature tolerance raw data

**Temperature (℃)**	**Triple Repeat Results**			**Average**	**Standard deviation**
4	6.85	6.70	6.78	6.77	0.05
37	6.60	6.81	6.74	6.72	0.08
45	6.78	6.54	6.60	6.64	0.09
55	6.70	6.95	6.30	6.65	0.23
65	6.30	6.00	6.00	6.10	0.13
70	3.52	3.30	3.70	3.51	0.14
80	1.48	1.30	0.30	1.03	0.48

Table S4 Phage pB3074 UV tolerance raw data

UV time(min)	Triple Repeat Results			Average	Standard deviation
0	6.85	6.75	6.60	6.73	0.09
10	4.30	3.90	4.70	4.30	0.27
20	3.00	3.00	3.85	3.28	0.38
30	2.85	1.69	3.70	2.74	0.70
40	1.70	2.00	2.30	2.00	0.20
50	0.00	0.70	0.30	0.33	0.24
60	0.00	0.30	0.00	0.10	0.13

Table S5 Phage pB3074 MOI raw data

MOI	Triple Repeat Results			Average	Standard deviation
0.0001	8.53	8.38	8.60	8.50	0.08
0.001	8.41	8.48	8.30	8.40	0.06
0.01	8.51	8.68	8.74	8.64	0.09
0.1	8.33	8.18	8.30	8.27	0.06
1	8.28	8.37	8.33	8.33	0.03
10	8.20	8.32	8.27	8.26	0.04
100	8.08	7.98	8.00	8.02	0.04
100	6.76	6.81	6.65	6.74	0.06
1000	2.78	2.70	2.78	2.75	0.04

Table S6 Phage pB3074 one step growth curve raw data

Time(min)	Triple Repeat Results			Average	Standard deviation
0	5.00	5.48	5.65	5.38	0.25
10	4.88	4.78	4.51	4.72	0.14
20	5.34	5.05	5.60	5.33	0.19
30	5.52	4.98	5.38	5.29	0.21
40	6.00	5.20	5.85	5.68	0.32
50	6.18	6.50	5.93	6.20	0.20
60	7.44	7.11	7.60	7.38	0.18
70	7.68	7.50	7.40	7.53	0.10
80	7.88	8.00	7.97	7.95	0.05
90	7.92	7.70	7.78	7.80	0.08
100	7.87	8.00	7.74	7.87	0.09
110	8.08	8.35	8.54	8.33	0.16
120	8.19	8.30	8.32	8.27	0.05

Table S7 Effect of pB3074 and antibiotics [cefotaxime(2×MIC) or meropenem (0.5×MIC)] combination on Bm3074 biofilm formation

**Group**	**Bacterial log concentration**					**Bacterial OD_{600}**				
	Triple Repeat Results			**Ave**	**SD**	Triple Repeat Results			**Ave**	**SD**
Bacteria	8.78	9.41	8.95	9.05	0.24	0.62	0.78	0.87	0.76	0.09
Bacteria+ Meropenem	8.30	9.48	9.18	8.98	0.46	0.68	0.71	0.75	0.71	0.02
Bacteria+ Cefotaxime	8.60	9.34	9.00	8.98	0.25	0.66	0.83	0.71	0.73	0.06
pB3074	8.70	8.60	8.40	8.57	0.11	0.55	0.64	0.81	0.67	0.10
Bacteria+Meropenem+pB3074	5.48	4.60	3.08	4.39	0.87	0.07	0.04	0.04	0.05	0.01
Bacteria+Cefotaxime+pB3074	7.08	7.48	6.60	7.05	0.30	0.14	0.10	0.08	0.11	0.02

Note: Ave: Average, SD: Standard deviation

Table S8 Effect of pB3074 and antibiotics [cefotaxime(2×MIC) or meropenem (0.5×MIC)] combination on Bm3074 mature biofilm-reducing

**Group**	**Bacterial log concentration**					**Bacterial OD_{600}**				
	Triple Repeat Results			**Ave**	**SD**	Triple Repeat Results			**Ave**	**SD**
Bacteria	9.70	8.00	9.18	8.96	0.64	0.99	0.71	0.88	0.86	0.10
Bacteria+ Meropenem	9.30	8.48	7.85	8.54	0.51	0.91	0.8	0.65	0.79	0.09
Bacteria+ Cefotaxime	8.78	8.30	7.98	8.35	0.28	0.84	0.75	0.6	0.73	0.09
pB3074	8.70	7.30	8.54	8.18	0.59	0.7	0.56	0.75	0.67	0.07
Bacteria+Meropenem+pB3074	4.48	2.41	3.88	3.59	0.78	0.05	0.04	0.07	0.05	0.01
Bacteria+Cefotaxime+pB3074	8.10	6.54	4.08	6.24	1.44	0.6	0.2	0.16	0.32	0.19

Note: Ave: Average, SD: Standard deviation

Table S9 Antibacterial effect of pB3074 alone or a combination with antibiotic [cefotaxime(2×MIC) or meropenem (0.5×MIC)] in an *ex vivo* model of wounded skin.

**Group**	**8 h**					**24 h**				
	Triple Repeat Results			Ave	SD	Triple Repeat Results			AVE	SD
Bacteria	9.70	9.60	9.18	9.49	0.21	0.86	0.83	0.85	0.85	0.01
Bacteria+ Meropenem	9.70	8.48	8.85	9.01	0.46	0.9	0.65	0.7	0.75	0.10
Bacteria+ Cefotaxime	9.00	8.90	8.74	8.88	0.09	0.85	0.79	0.68	0.77	0.06
pB3074	8.00	7.85	8.54	8.13	0.28	0.55	0.48	0.61	0.55	0.04
Bacteria+Meropenem+pB3074	6.48	5.41	5.88	5.92	0.37	0.12	0.09	0.15	0.12	0.02
Bacteria+Cefotaxime+pB3074	7.60	6.78	6.54	6.97	0.42	0.11	0.2	0.16	0.16	0.03

Note: Ave: Average, SD: Standard deviation

Table S10 the results of the host range determinations

No.	Plaque
A. baumannii 1113	++
A. baumannii 4211	+++
A. baumannii 1130	-
A. baumannii 3041	-
A. baumannii LB8	-
A. baumannii 4015	-
A. baumannii 4143	-
A. baumannii 4144	-
A. baumannii 2101	+++
A. baumannii 4142	+++
A. baumannii 1112	-
A. baumannii 1131	-
A. baumannii 0125	+++
A. baumannii 4141	+++
A. baumannii 3241	+++
A. baumannii 0025	+++
A. baumannii 005	+
A. baumannii 0122	-
A. baumannii 3076	-
A. baumannii 0111	-
A. baumannii 0128	-
A. baumannii 0210	-
A. baumannii 008	-
A. baumannii 1215	+++
A. baumannii 1216	
A. baumannii 1228	
A. baumannii 3305	+++
A. baumannii 3306	++
A. baumannii 3431	-
A. baumannii 0025	-
A. baumannii 004	+
A. baumannii 4341	+++
A. baumannii 3451	-
A. baumannii 3033	-
Escherichia coli	-
Pseudomonas aeruginosa	-
Streptococcus pneumoniae	-

Note: +++, ++ and+ mean very clear, clear and faint of the plaques, respectively. - means no lysis plaque.

Table S11 annotated genes

Gene_ID	Gene_Length	Gene_description	Gene_Start	Gene_End	Gene_Strand
PROKKA_00001	663	putative internal virion protein B	1117	1779	-
PROKKA_00002	2292	tail tubular protein B	1791	4082	-
PROKKA_00003	627	tail tubular protein A	4091	4717	-
PROKKA_00004	186	hypothetical protein	4830	5015	-
PROKKA_00005	1032	putative capsid protein	5071	6102	-
PROKKA_00006	861	putative scaffolding protein	6118	6978	-
PROKKA_00007	1557	putative head-tail connector protein	6987	8543	-
PROKKA_00008	252	structural protein	8552	8803	-
PROKKA_00009	198	hypothetical protein	8800	8997	-
PROKKA_00010	2418	phage-associated RNA polymerase	9101	11518	-
PROKKA_00011	654	putative dNMP kinase	11527	12180	-
PROKKA_00012	936	hypothetical protein	12180	13115	-
PROKKA_00013	441	putative DNA endonuclease VII	13119	13559	-
PROKKA_00014	570	hypothetical protein	13556	14125	-
PROKKA_00015	966	putative DNA exonuclease	14115	15080	-
PROKKA_00016	333	hypothetical protein	15061	15393	-
PROKKA_00017	891	hypothetical protein	15566	16456	-
PROKKA_00018	240	hypothetical protein	16460	16699	-
PROKKA_00019	939	putative DNA polymerase	16689	17627	-
PROKKA_00020	528	HNH endonuclease	17713	18240	-
PROKKA_00021	1356	putative DNA polymerase	18337	19692	-
PROKKA_00022	978	putative ATP-dependent DNA ligase	19941	20918	-
PROKKA_00023	744	-	20915	21658	-
PROKKA_00024	1299	putative DNA helicase	21655	22953	-
PROKKA_00025	243	hypothetical protein	22966	23208	-
PROKKA_00026	318	hypothetical protein	23208	23525	-

PROKKA_00027	315	-	23525	23839	-
PROKKA_00028	801	putative DNA primase	23842	24642	-
PROKKA_00029	267	hypothetical protein	24632	24898	-
PROKKA_00030	219	hypothetical protein	24840	25058	-
PROKKA_00031	192	hypothetical protein	25055	25246	-
PROKKA_00032	168	hypothetical protein	25233	25400	-
PROKKA_00033	438	hypothetical protein	25411	25848	-
PROKKA_00034	501	hypothetical protein	25850	26350	-
PROKKA_00035	399	hypothetical protein	26421	26819	-
PROKKA_00036	105	hypothetical protein	26978	27082	-
PROKKA_00037	597	hypothetical protein	27079	27675	-
PROKKA_00038	375	hypothetical protein	27854	28228	-
PROKKA_00039	510	hypothetical protein	28230	28739	-
PROKKA_00040	471	-	28810	29280	-
PROKKA_00041	264	-	30094	30357	-
PROKKA_00042	180	-	31097	31276	-
PROKKA_00043	204	hypothetical protein	31287	31490	-
PROKKA_00044	138	hypothetical protein	31447	31584	-
PROKKA_00045	1938	putative DNA maturase B	31581	33518	-
PROKKA_00046	309	hypothetical protein	33528	33836	-
PROKKA_00047	558	EF hand domain protein	33897	34454	-
PROKKA_00048	336	hypothetical protein	34441	34776	-
PROKKA_00049	2082	-	34790	36871	-
PROKKA_00050	3099	putative internal virion protein	36878	39976	-
PROKKA_00051	1890	hypothetical protein	39986	41875	-

Table S12 Accession number of phage used in phylogenetic tree

Phage name in Tree	Accession number
Acinetobacter phage Ab105-1phi	KT588074.1
Acinetobacter phage YMC11	NC_041866.1
Acinetobacter phage YC#06	ON391949.1
Enterobacteria phage K30	NC_015719.1
Acinetobacter phage YMC13/03/R2096	NC_027332.1
Acinetobacter bacteriophage AP22	NC_017984.1
Acinetobacter phage AB3	NC_021337.1
**PB3074**	BankIt2690067 pB3074 OQ730192
Acinetobacter phage Abp1	NC_021316.1
Acinetobacter phage SH-Ab 15519	NC_041905.1

A																	
	Time:h	Bacteria		Bacteria+Piperacillin		Bacteria+Phage(L)		Bacteria+Phage(M)		Bacteria+Phage(H)		acteria+Piperacillin+Phage(l		Bacteria+Piperacillin+Phage(M)		Bacteria+Piperacillin+Phage(H)	
		Ave	SD	Ave	SD	Ave	SD	Ave	SD	Ave	SD	Ave	SD	Ave	SD	Ave	SD
	0	5.94	0.24	5.94	0.24	5.94	0.24	5.94	0.24	5.94	0.24	5.94	0.24	5.94	0.24	5.94	0.24
	8	8.34	0.26	7.89	0.11	5.24	0.26	5.04	0.35	1.50	0.50	1.35	0.65	0.85	0.70	0.50	0.50
	16	9.32	0.06	8.74	0.26	7.80	0.20	7.91	0.91	8.19	0.19	7.28	0.50	6.47	0.17	7.14	0.06
	24	9.19	0.19	9.05	0.15	8.64	0.16	8.87	0.09	9.03	0.05	8.17	0.17	6.95	0.17	8.15	0.15
	48	9.25	0.35	8.69	0.15	8.54	0.06	8.39	0.48	8.66	0.18	8.34	0.34	7.78	0.15	8.39	0.39
B	Time:h	Bacteria		Bacteria+Ceftazidime		Bacteria+Phage(L		Bacteria+Phage(		Bacteria+Phage(H)		Bacteria+Ceftazidime+l		Bacteria+Ceftazidime+Phaş		Bacteria+Ceftazidime+Phage(H)	
		Ave	SD	Ave	SD	Ave	SD	Ave	SD	Ave	SD	Ave	SD	Ave	SD	Ave	SD
	0	5.94	0.24	5.94	0.24	5.94	0.24	5.94	0.24	5.94	0.24	5.94	0.24	5.94	0.24	5.94	0.24
	8	8.34	0.26	8.00	0.50	5.24	0.26	5.04	0.35	1.50	0.50	1.10	0.60	0.56	0.45	0.00	0.00
	16	9.32	0.06	9.00	0.33	7.80	0.20	7.91	0.91	8.19	0.19	5.35	0.35	5.19	0.41	6.54	0.24
	24	9.19	0.19	9.21	0.55	8.64	0.16	8.87	0.09	9.03	0.05	6.22	0.62	5.25	0.05	8.34	0.04
	48	9.25	0.35	8.43	0.42	8.54	0.06	8.39	0.48	8.66	0.18	6.43	0.53	6.20	0.18	8.24	0.54
C	Time:h	Bacteria		Bacteria+Cefotaxime		Bacteria+Phage(L		Bacteria+Phage(		Bacteria+Phage(H)		Bacteria+Cefotaxime+l		Bacteria+Cefotaxime+Phag		Bacteria+Cefotaxime+Phage(H)	
		Ave	SD	Ave	SD	Ave	SD	Ave	SD	Ave	SD	Ave	SD	Ave	SD	Ave	SD
	0	5.94	0.24	5.94	0.24	5.94	0.24	5.94	0.24	5.94	0.24	5.94	0.24	5.94	0.24	5.94	0.24
	8	8.34	0.26	7.50	0.25	5.24	0.26	5.04	0.35	1.50	0.50	5.89	0.11	0.00	0.00	3.65	0.35
	16	9.32	0.06	8.65	0.55	7.80	0.20	7.91	0.91	8.19	0.19	7.30	0.30	4.89	0.59	7.13	0.83
	24	9.19	0.19	9.00	0.35	8.64	0.16	8.87	0.09	9.03	0.05	8.22	0.22	6.34	1.14	8.69	0.09
	48	9.25	0.35	8.60	0.25	8.54	0.06	8.39	0.48	8.66	0.18	7.84	0.24	5.61	0.36	8.34	0.26
D	Time:h	Bacteria		Bacteria+Imipenem		Bacteria+Phage(L		Bacteria+Phage(		Bacteria+Phage(H)		Bacteria+Imipenem+Pl		Bacteria+Imipenem+Phage		Bacteria+Imipenem+Phage(H)	
		Ave	SD	Ave	SD	Ave	SD	Ave	SD	Ave	SD	Ave	SD	Ave	SD	Ave	SD
	0	5.94	0.24	5.94	0.24	5.94	0.24	5.94	0.24	5.94	0.24	5.94	0.24	5.94	0.24	5.94	0.24
	8	8.34	0.26	8.10	0.34	5.24	0.26	5.04	0.35	1.50	0.50	0.00	0.00	0.00	0.00	0.00	0.00
	16	9.32	0.06	9.27	0.21	7.80	0.20	7.91	0.91	8.19	0.19	5.93	0.45	0.00	0.00	0.00	0.00
	24	9.19	0.19	9.10	0.65	8.64	0.16	8.87	0.09	9.03	0.05	7.69	0.31	0.00	0.00	1.25	1.00
	48	9.25	0.35	8.90	0.25	8.54	0.06	8.39	0.48	8.66	0.18	8.19	0.19	0.00	0.00	7.00	0.30
E	Time:h	Bacteria		Bacteria+Meropenem		Bacteria+Phage(L		Bacteria+Phage(		Bacteria+Phage(H)		Bacteria+Meropenem+		Bacteria+Meropenem+Pha		Bacteria+Meropenem+Phage(H)	
		Ave	SD	Ave	SD	Ave	SD	Ave	SD	Ave	SD	Ave	SD	Ave	SD	Ave	SD
	0	5.94	0.24	5.94	0.24	5.94	0.24	5.94	0.24	5.94	0.24	5.94	0.24	5.94	0.24	5.94	0.24
	8	8.34	0.26	7.95	0.24	5.24	0.26	5.04	0.35	1.50	0.50	0.00	0.00	0.00	0.00	5.15	0.75
	16	9.32	0.06	8.95	0.65	7.80	0.20	7.91	0.91	8.19	0.19	4.89	0.11	1.65	0.65	5.93	0.15
	24	9.19	0.19	9.45	0.45	8.64	0.16	8.87	0.09	9.03	0.05	7.99	0.09	5.80	0.20	8.10	0.10
	48	9.25	0.35	8.50	0.55	8.54	0.06	8.39	0.48	8.66	0.18	8.22	0.38	6.39	0.51	8.55	0.25
F	Time:h	Bacteria		Bacteria+Aztreonam		Bacteria+Phage(L		Bacteria+Phage(		Bacteria+Phage(H)		Bacteria+Aztreonam+P		Bacteria+Aztreonam+Phag		Bacteria+Aztreonam+Phage(H)	
		Ave	SD	Ave	SD	Ave	SD	Ave	SD	Ave	SD	Ave	SD	Ave	SD	Ave	SD
	0	5.94	0.24	5.94	0.24	5.94	0.24	5.94	0.24	5.94	0.24	5.94	0.24	5.94	0.24	5.94	0.24
	8	8.34	0.26	8.35	0.25	5.24	0.26	5.04	0.35	1.50	0.50	3.74	0.26	1.15	0.15	0.00	0.00
	16	9.32	0.06	9.20	0.45	7.80	0.20	7.91	0.91	8.19	0.19	6.24	0.54	0.00	0.00	3.30	0.95
	24	9.19	0.19	9.60	0.25	8.64	0.16	8.87	0.09	9.03	0.05	6.69	0.15	0.00	0.00	7.69	0.39
	48	9.25	0.35	8.45	0.44	8.54	0.06	8.39	0.48	8.66	0.18	6.75	0.45	0.00	0.00	7.81	0.33

FIGS1 Data of effect of phage dose on PAS between pB3074 and antibiotics including piperacillin(A), ceftazidime(B), cefotaxime(C), imipenem(D), meropenem(E) and aztreonam(F), in combination.

Note: Ave: Average, SD: Standard deviation

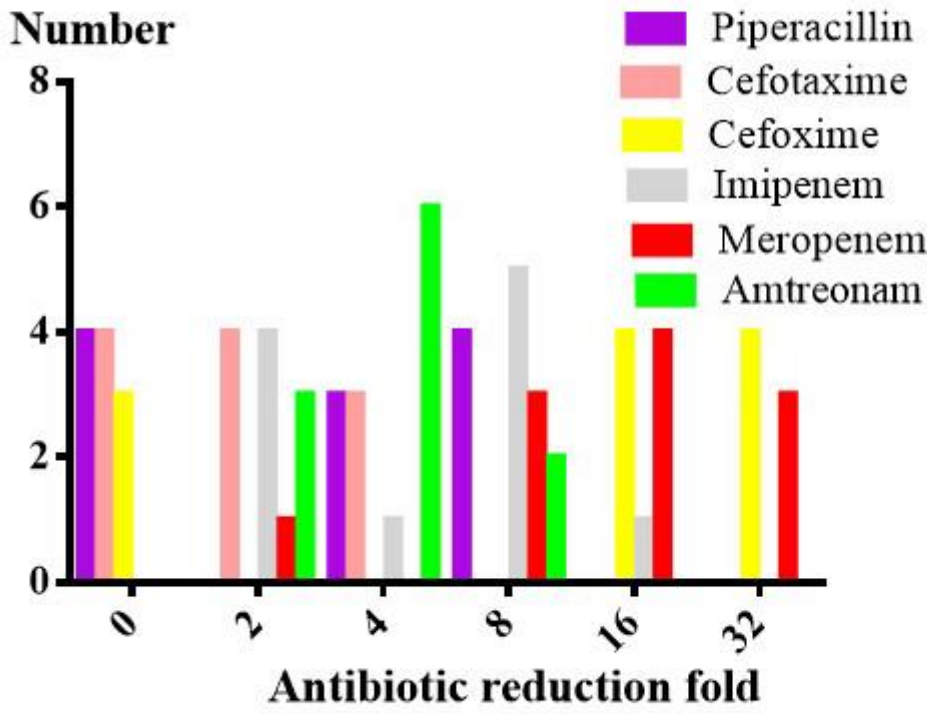

FIGS2 Number statistic of antibiotic concentration reduction fold

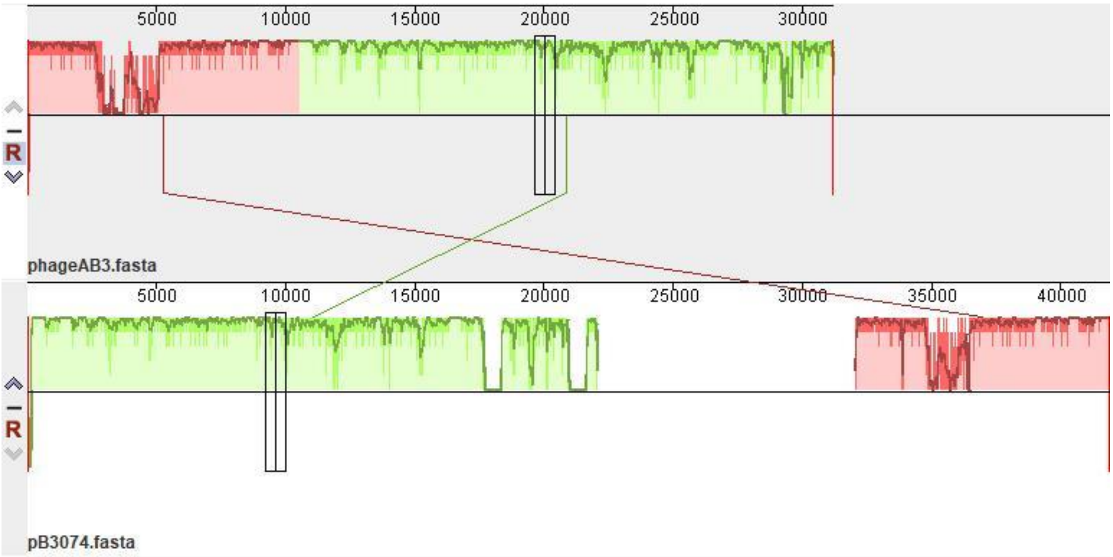

FIGS3 Difference between phage AB3 and pB3074
